# Dose-response association between dietary folate and niacin intakes with diabetes among Chinese adults: a cross-sectional study

**DOI:** 10.1186/s41043-023-00362-w

**Published:** 2023-04-10

**Authors:** Yuhong Jiang, Zhiwen Zhang, Yi Zhu, Yongfei Chai, Hong Xie

**Affiliations:** 1 Department of Epidemiology and Health Statistics, School of Public Health, Bengbu Medical College, Bengbu, China; 2 School of Public Health, Bengbu Medical College, Bengbu, China; 3 Department of Nutrition and Food Hygiene School of Public Health, Bengbu Medical College, Bengbu, China

**Keywords:** Dietary intake, Vitamins, Diabetes, Middle-aged Chinese

## Abstract

**Background:**

The aim of this study was to examine the relationship between dietary intake of folate and niacin and diabetes risk in Chinese adults.

**Methods:**

This is a cross-sectional study. Demographic and anthropometric data along with information on dietary intake of vitamins were collected, and eligible participants were recruited to complete the questionnaire. A binary logistic regression analysis was conducted to examine the association between dietary intake of vitamins and diabetes risk, with adjustment for potential confounders. Non-linear dose-response relationships between dietary intake of folate and niacin and diabetes risk were also evaluated using adjusted restricted cubic splines.

**Results:**

Of the 3106 eligible participants, 15.9% had diabetes. Median folate was significantly higher in diabetic patients than in controls (32.030 vs. 27.600 gμ), while median niacin was significantly lower (7.000 vs. 7.900 mg). After controlling for potential confounders, binary logistic regression analysis showed that each unit increase in folate intake was associated with a 1.002-fold increase in the risk of developing diabetes (odds ratio (OR) = 1.002; 95% confidence interval (CI) 1.000–1.004; *P* = 0.022), while each unit increase in niacin intake was associated with a 3.5% reduction in diabetes risk (OR = 0.965; 95% CI 0.944–0.986; *P* = 0.001). The plots of restricted cubic splines presented an atypical inverted U-shaped association between dietary intake of folate and diabetes risk.

**Conclusions:**

Diabetic patients had a low intake of vitamins, especially the B vitamins. Dietary intake of folate and niacin tended to be independently associated with the risk of diabetes. Nevertheless, this study is observational and a large-scale randomized controlled trial is yet to be conducted, which will add to the evidence of the study results.

## Background

Diabetes is one of the fastest growing health challenges of the twenty-first century, with the number of adults with diabetes more than tripling in the last 20 years [1]. Diabetes has the potential to cause numerous debilitating health complications and increase the risk of early death [1]. Investing in effective diabetes prevention and management has become necessary to prevent disability and death.

It is well known that diabetes is recognized as a multifactorial chronic disease that can be related to dietary factors [2]. Economic growth and environmental transitions have led to drastic changes in food production, processing, and distribution systems, increasing the accessibility of unhealthful foods [3]. With the nutritional transition, people are experiencing increased morbidity and mortality from diabetes. [4]

Several epidemiological studies [2, 5, 6] have shown that diet plays an important role in the development of diabetes and have reported an association between nutrient intake and diabetes risk [5, 6]. In addition, diabetic patients should be informed about the importance of acquiring daily vitamin requirements through a well-balanced diet, as micronutrient deficiencies are often present in individuals with poorly controlled diabetes [7]. Adequate intake of B vitamins is a general requirement for healthy cell growth and nucleic acid synthesis in all cells. A few studies [8–15] reported that B vitamins, especially folate (vitamin B9) and niacin (vitamin B3), may be involved in the pathogenesis of glucose intolerance and are inversely associated with diabetes risk. However, these findings are inconsistent. Furthermore, most previous studies have addressed vitamin status [16, 17] or supplementation [18–20], rather than dietary intake [10, 11].

Therefore, it is of interest to explore the dietary vitamin intake and its relationship with diabetes risk. In view of this, this study aimed to characterize the dietary vitamin intake of Chinese adults and to evaluate the association between dietary folate and niacin intake and diabetes risk in the hope of providing a scientific rationale for formulating dietary guidelines.

## Methods

### Study population

A multi-stage random sampling design was used in Bengbu, China, to investigate the epidemiological characteristics of major chronic noncommunicable diseases among residents living in the community for more than 6 months [21]. Individuals with severe mental illness or cognitive impairment and those who were pregnant or breastfeeding were excluded. This cross-sectional study was reviewed and approved by the Ethics Committee of Bengbu medical college. All participants were required to complete the entire survey and sign an informed consent form.

### Data collection

Demographic information was obtained through questionnaires by trained staff. Standardization of techniques and staff training were conducted prior to the start of the survey to reduce inter-observer variation. Gender (male and female), age (years), educational level (elementary level or lower, junior high school level, high school level, and college level or higher), marital status (unmarried, currently married, and other), place of residence (urban and rural), and smoking (current and non-smoker) were collected.

Anthropometric data were collected by trained staff with uniform instruments. The staff received instruction and training from professional physicians before performing the measurements. Each subject’s height (m) and weight (kg) were measured wearing light indoor clothing. Body mass index (BMI) was calculated by dividing weight by the square of height, and BMI ≥ 28 kg/m^2^ was set as general obesity [22]. For waist circumference (WC) measurements, subjects should be in a fasted and upright position to avoid measurement errors due to eating and body position. Waist circumference ≥ 90 cm in men and ≥ 85 cm in women were considered as abdominal obesity, respectively [23].

Blood samples were collected after an overnight fast of more than 8 h to estimate fasting plasma glucose (FPG) and glycosylated hemoglobin (HbA1c) values. The day before the blood draw, the community physician carried out relevant publicity and mobilization in the community and asked again about the feeding time before drawing blood. The collected blood samples would be sent to the laboratory of the affiliated hospital to have the results tested.

### Assessment of diabetes

The diagnosis of diabetes in this study was based on participants’ self-reports. The investigators asked “Have you ever been diagnosed with diabetes by a doctor?” to confirm whether the participants had diabetes.

### Assessment of vitamin intakes

A three-day food record was used in assessing dietary vitamin intake. Prior to the survey, to maintain the accuracy of the food record data, investigators made several visits to the community to promote the survey and were trained by a medical professional on how to obtain the food records. The types and amounts of food collected were entered into the Food Nutrition Calculator V2.65, developed and recommended by the National Institute of Nutrition and Food Safety of the Chinese Center for Disease Control and Prevention, which automatically generated and derived the required vitamin levels.

### Statistical analyses

The Kolmogorov–Smirnov test was applied to verify the normality of the data. Data for continuous variables were expressed as medians (interquartile range) and for categorical variables as numbers (percentages). The Mann–Whitney U test and chi-square test were used to compare the medians of continuous variables and the percentages of categorical variables between the diabetic and non-diabetic groups, respectively. After adjusting for potential confounding variables, the odds ratios (ORs) with 95% confidence intervals (95% CIs) for the association between dietary vitamin intake and diabetes risk were calculated for each unit of vitamin intake using a binary logistic regression model. Tolerance (TOL) and variance inflation factor (VIF) were used to detect collinearity between variables included in the regression models, with TOL < 0.1 and VIF > 10 considered indicative of collinearity [24]. In the fully adjusted model, restricted cubic splines with four knots at the 25th, 50th, 75th, and 95th percentiles of the exposure distribution were used to flexibly establish the relationship between dietary folate and niacin intake and diabetes risk. All statistical analyses were undertaken using SPSS 23.0 (IBM, Armonk, NY, USA) and Stata 12.0 software (StataCorp., College Station, TX, USA). *P* < 0.05 was considered to be statistically significant.

## Results

### Characteristics of the study population

A total of 3115 participants were recruited. Excluding 9 individuals who did not complete the questionnaire regarding dietary vitamin intake, 3106 individuals were eventually included. The prevalence of diabetes was 15.9%, with 19.0% in men and 13.7% in women. The median age of the diabetic patients (64 years) was higher than that of the non-diabetic control group (57 years, *P* < 0.001). A significantly higher proportion of general obesity (χ^2^ = 18.914, *P* < 0.001) and abdominal obesity (χ^2^ = 58.254, *P* < 0.001) were found in the diabetic group compared to the non-diabetic group (Tables 1 and 2).

**Table 1:** Characteristics of participants grouped by diabetes

Variables	Non-diabetic group (n = 2611)	Diabetic group (n = 495)	*P* value
Female, n (%)	1556 (59.6)	247 (49.9)	< 0.001
Age (year)	57 (45, 66)	64 (56, 72)	< 0.001
Educational level, n (%)			< 0.001
Elementary level or lower	735 (28.2)	187 (37.9)	
Junior high school level	963 (37.0)	170 (34.4)	
High school level	646 (24.8)	99 (20.0)	
College level or higher	258 (9.9)	38 (7.7)	
Marital status, n (%)			0.003
Unmarried	92 (3.5)	6 (1.2)	
Currently married	2198 (84.8)	411 (83.5)	
Others (widowed, divorced, etc.)	302 (11.7)	75 (15.2)	
Place of residence, n (%)			0.004
Urban	2156 (82.6)	435 (87.9)	
Rural	455 (17.4)	60 (12.1)	
Smoking, n (%)	734 (28.1)	174 (35.2)	0.002
General obesity, n (%)	343 (13.1)	102 (20.6)	< 0.001
Abdominal obesity, n (%)	1036 (39.7)	288 (58.2)	< 0.001
FPG (mmol/L)	4.82 (4.36, 5.33)	7.40 (6.29, 9.52)	< 0.001
HbA1c (%)	4.90 (4.30, 5.50)	7.10 (5.80, 8.40)	< 0.001
Energy (kcal)	1408.00 (1129.00, 1739.00)	1372.00 (1144.00, 1673.00)	0.499
Vitamin A (μgRE)	280.00 (134.00, 500.00)	285.00 (155.00, 435.00)	0.946
Thiamine (mg)	0.44 (0.32, 0.64)	0.43 (0.31, 0.56)	0.024
Riboflavin (mg)	0.59 (0.42, 0.81)	0.56 (0.42, 0.73)	0.018
Vitamin B6 (mg)	0.12 (0.05, 0.22)	0.12 (0.06, 0.21)	0.307
Folate (μg)	27.60 (9.30, 57.80)	32.03 (15.37, 57.70)	0.001
Niacin (mg)	7.90 (5.50, 11.60)	7.00 (5.00, 9.40)	< 0.001
Vitamin C (mg)	45.70 (23.30, 79.50)	40.60 (25.30, 62.80)	0.010
Vitamin E (mg)	24.75 (14.95, 41.73)	27.82 (16.39, 42.19)	0.046

General obesity, Body mass index (BMI) ≥ 28 kg/m^2^; Abdominal obesity, male: waist circumference (WC) ≥ 90 cm and female: WC ≥ 85 cm; FPG, fasting plasma glucose; HbA1c, glycosylated hemoglobin

**Table 2:** Dietary vitamin intakes between diabetic and non-diabetic group stratified by gender

Variables	Male			Female		
	Non-diabetic group (n = 1055)	Diabetic group (n = 248)	*P* value	Non-diabetic group (n = 1556)	Diabetic group (n = 247)	*P* value
Vitamin A (μgRE)	285.00 (143.00, 522.00)	304.50 (160.25, 454.75)	0.852	274.50 (127.25, 484.00)	275.00 (149.00, 407.00)	0.739
Thiamine (mg)	0.48 (0.34, 0.71)	0.48 (0.34, 0.60)	0.164	0.42 (0.30, 0.59)	0.40 (0.28, 0.50)	0.005
Riboflavin (mg)	0.62 (0.45, 0.88)	0.57 (0.45, 0.77)	0.064	0.57 (0.40, 0.78)	0.53 (0.40, 0.70)	0.026
Vitamin B6 (mg)	0.12 (0.06, 0.24)	0.13 (0.07, 0.21)	0.956	0.10 (0.04, 0.21)	0.11 (0.06, 0.19)	0.355
Folate (μg)	30.80 (11.30, 64.90)	35.73 (17.76, 57.12)	0.154	25.25 (8.20, 54.75)	28.47 (13.80, 58.20)	0.011
Niacin (mg)	9.00 (6.20, 13.60)	7.80 (5.65, 10.23)	< 0.001	7.20 (5.10, 10.50)	6.27 (4.67, 8.30)	< 0.001
Vitamin C (mg)	45.10 (22.50, 78.70)	41.65 (25.42, 64.87)	0.295	46.20 (23.82, 80.07)	38.90 (25.20, 61.40)	0.011
Vitamin E (mg)	26.21 (15.85, 44.14)	28.42 (16.05, 43.48)	0.668	23.84 (14.53, 40.10)	26.36 (16.62, 40.11)	0.035

### Regression analysis

After adjusting for gender, age, educational level, marital status, place of residence, smoking, general obesity, abdominal obesity, and energy intake, binary logistic regression analysis showed that each unit increase in folate intake was associated with a 1.002-fold increase in the likelihood of developing diabetes (OR = 1.002; 95% CI 1.000–1.004; *P* = 0.022), while each unit increase in niacin intake was associated with a 3.5% reduction in the risk of developing diabetes (OR = 0.965; 95% CI 0.944–0.986; *P* = 0.001).

In adjusted regression models stratified by gender, each unit increase in folate intake was associated with a 1.004-fold increase in the risk of diabetes in women (OR = 1.004; 95% CI 1.001–1.006; *P* = 0.002). However, for each unit increase in niacin intake, the risk of developing diabetes was reduced by 3% in men (OR = 0.970; 95% CI 0.945–0.996; *P* = 0.024).

No significant collinearity was found in all regression models based on the values of TOL and VIF (Tables 3, 4 and 5).

**Table 3:** Predictors of diabetes using the binary logistic regression analysis

Variables	OR (95% CI)	*P* value	Collinearity	
			TOL	VIF
Gender (Female)	0.657 (0.536, 0.805)	< 0.001	0.575	1.740
Age (year)	1.037 (1.029, 1.045)	< 0.001	0.681	1.468
WC (Abdominal obesity)	1.840 (1.503, 2.252)	< 0.001	0.829	1.206
Folate (μg)	1.002 (1.000, 1.004)	0.022	0.648	1.543
Niacin (mg)	0.965 (0.944, 0.986)	0.001	0.383	2.611

OR, Odds ratio; CI, confidence interval; TOL, Tolerance; VIF, Variance Inflation Factor

**Table 4:** Predictors of diabetes in male participants

Variables	OR (95% CI)	*P* value	Collinearity	
			TOL	VIF
Age (year)	1.024 (1.013, 1.035)	< 0.001	0.707	1.415
Place of residence (Urban)	0.569 (0.362, 0.895)	0.015	0.780	1.281
Abdominal obesity	1.378 (1.035, 1.834)	0.028	0.849	1.178
Niacin (mg)	0.970 (0.945, 0.996)	0.024	0.372	2.690

OR, Odds ratio; CI, confidence interval; TOL, Tolerance; VIF, Variance Inflation Factor

**Table 5:** Predictors of diabetes in female participants

Variables	OR (95% CI)	*P* value	Collinearity	
			TOL	VIF
Age (year)	1.052 (1.040, 1.064)	< 0.001	0.651	1.535
General obesity	1.458 (1.015, 2.096)	0.041	0.855	1.170
Abdominal obesity	2.089 (1.532, 2.848)	< 0.001	0.804	1.244
Folate (μg)	1.004 (1.001, 1.006)	0.002	0.613	1.631

OR, Odds ratio; CI, confidence interval; TOL, Tolerance; VIF, Variance Inflation Factor

### Restricted cubic spline

**Fig. 1:**
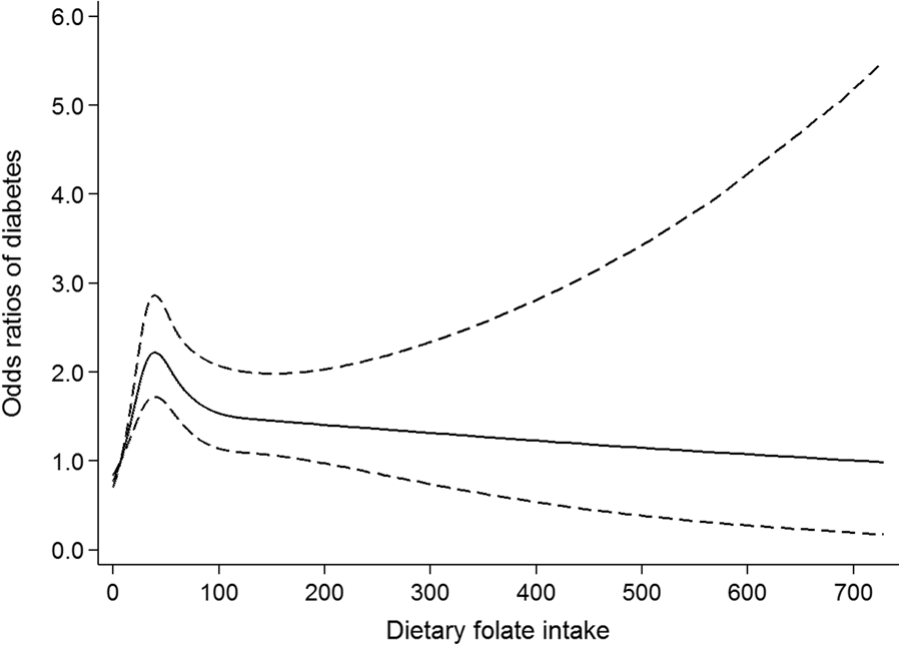
Restricted cubic spline model of the odds ratios of diabetes with dietary folate intake. Adjusted for gender, age, educational level, marital status, place of residence, smoking, general obesity, abdominal obesity and energy intake. The dashed lines represent the 95% confidence intervals

**Fig. 2:**
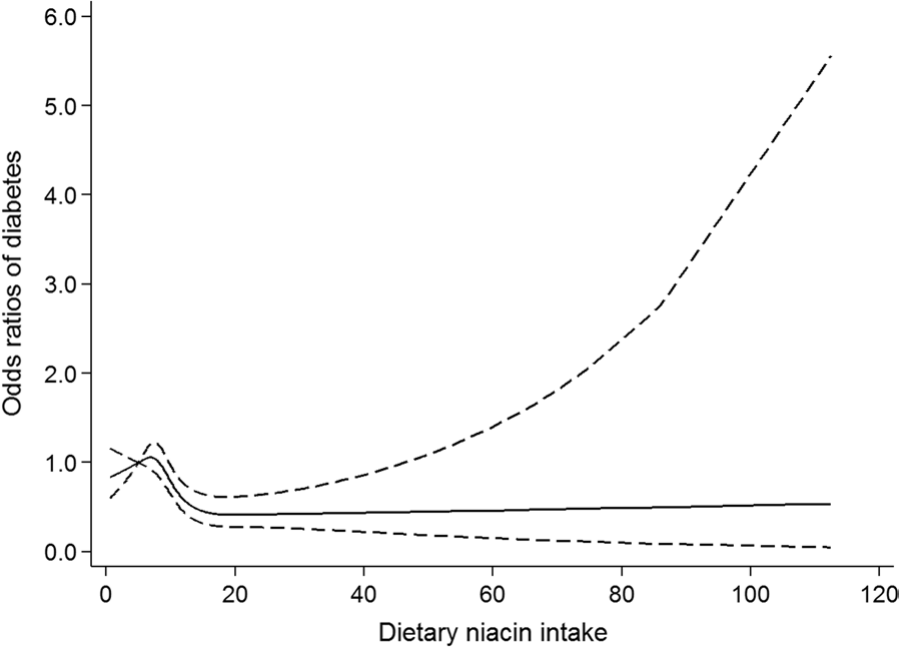
Restricted cubic spline model of the odds ratios of diabetes with dietary niacin intake. Adjusted for gender, age, educational level, marital status, place of residence, smoking, general obesity, abdominal obesity and energy intake. The dashed lines represent the 95% confidence intervals

The results of the restricted cubic spline analysis were depicted in Figs. 1 and 2. After adjusting for potential confounders, the risk of developing diabetes increased with increasing folate intake until around 41.6 gμ and then started to decrease. While the risk of developing diabetes increased with increasing intake of niacin until around 7.2 mg, then showed a decrease until 27.6 mg, then increased again.

## Discussion

The prevalence of diabetes reported in this study was 15.9%. Diabetic patients had significantly higher dietary folate intake than controls, whereas dietary niacin intake was significantly lower than controls. After adjusting for potential confounders, dietary folate intake was positively associated with diabetes risk, whereas niacin intake was inversely associated with diabetes risk. In addition, the plots of restricted cubic spline presented an inverted U-shaped association between dietary folate intake and diabetes risk. There was a significant difference in folate intake in women and niacin intake in men between the diabetic and non-diabetic groups according to gender.

The B vitamins are essential water-soluble nutrients. Folate (vitamin B9) can be found in a wide variety of foods, such as vegetables, fruits, and nuts [25]. Two previous prospective cohort studies reported that dietary intake of folate was inversely associated with incident diabetes in Korean or Japanese women [10, 11]. Diabetes is an oxidative stress disease manifested by low serum glutathione levels, impaired total antioxidant status and antioxidant enzymatic activity [26]. Folate deficiency has been linked to oxidative stress in diabetic patients, in relation to a resulting hyperhomocysteinemia [8, 26]. Folate deficiency has also been reported to severely impede insulin biosynthesis and secretion from pancreatic β-cells [27]. However, this study showed that dietary folate intake was higher in diabetic patients than in controls. The anti-diabetic drug metformin may contribute to folate deficiency. In a randomized controlled trial (RCT), diabetic men taking metformin showed an improvement in total serum antioxidant capacity after 8 weeks of folate supplementation [28]. Therefore, it is possible that the higher folate intake in diabetic patients is due to reverse causality. Diabetic patients may have been advised to change their dietary habits during routine examinations in the past.

Niacin (vitamin B3) can be found in meat (especially liver and heart), fish, nuts and some fruits and vegetables, as well as in coffee [29]. Niacin is known to reduce triglyceride and low-density lipoprotein cholesterol levels while significantly increasing high-density lipoprotein cholesterol levels [30]. These lipid-modifying effects may play a role in diabetes-induced atherosclerosis [8]. In addition to lipid-modification, niacin has been reported to reduce monocyte adhesion, which is an important process in the development of atherosclerosis in diabetic patients [31]. Few studies have evaluated the relationship between dietary niacin intake and diabetes. Inadequate intake of niacin from food may not yet be considered a problem. The present study indicated that individuals with low niacin intake had a high likelihood of developing diabetes. However, a Japanese study concluded that dietary intake of niacin was not associated with a reduced risk of diabetes [11]. An RCT study [13] showed that 3 years of niacin use in subjects with normal baseline blood glucose levels was associated with increased blood glucose levels and the risk of impaired fasting glucose. In contrast, Sazonov et al. [14] reported that the negative effects of niacin on blood glucose were clinically insignificant. However, further studies are needed regarding the relationship between dietary niacin intake and the risk of diabetes.

Several potential limitations warrant mention. First, this study was a cross-sectional design, which did not allow to establish the temporality of cause-effect relationship with certainty. Second, information on the use of glucose-lowering medications or vitamin supplements was not investigated, which may have influenced the results. Third, vitamin intake data were obtained from a three-day food record, which does not accurately reflect individual dietary intake due to faulty memory and underreporting. Finally, the study sample was recruited in Bengbu city and may not be representative of the entire general population, which may limit the generalizability of our findings. Nevertheless, to improve the reliability of our results, we adjusted for a number of known and proposed potential confounders in binary logistic regression models and restricted cubic splines analyses, and our findings add to the limited data on the association between dietary folate and niacin intake and diabetes risk in Chinese populations.

## Conclusions

The dose-response relationship between dietary folate and niacin intake and diabetes risk suggests that these vitamin-rich diets are important for the management of diabetes. Because vitamin intake is a simple and feasible indicator of diabetes, increasing intake of B vitamins in adults may be a cost-effective strategy for improving diabetes. Larger longitudinal and interventional studies are needed to quantify the preventive and therapeutic levels of dietary vitamin intake, particularly folate and niacin, for the management of diabetes in clinical practice.

## Data Availability

The datasets used and/or analysed during the current study are available from the corresponding author on reasonable request.

## Abbreviations

OR: Odds ratio
CI: Confidence interval
BMI: Body mass index
WC: Waist circumference
FPG: Fasting plasma glucose
HbA1c: Glycosylated hemoglobin
TOL: Tolerance
VIF: Variance inflation factor
RCT: Randomized controlled trial
